# Change in kidney volume after kidney transplantation in patients with autosomal polycystic kidney disease

**DOI:** 10.1371/journal.pone.0209332

**Published:** 2018-12-27

**Authors:** Massimiliano Veroux, Cecilia Gozzo, Daniela Corona, Paolo Murabito, Daniele Carmelo Caltabiano, Luca Mammino, Alessia Giaquinta, Domenico Zerbo, Nunziata Sinagra, Pierfrancesco Veroux, Stefano Palmucci

**Affiliations:** 1 Vascular Surgery and Organ Transplant Unit, Azienda Ospedaliera Policlinico of Catania, Catania, Italy; 2 Department of Medical Biopathology and Biotechnologies, University of Palermo, Palermo, Italy; 3 Anaesthesiology and Intensive Care Unit Azienda Ospedaliera Policlinico of Catania, Catania, Italy; 4 Radiology Unit, Azienda Ospedaliera Policlinico of Catania, Catania, Italy; University of Catania, ITALY

## Abstract

**Background:**

The indication to bilateral nephrectomy in patients with autosomal dominant polycystic kidney scheduled for kidney transplantation is controversial. Indeed, the progressive enlargement of cysts may increase the risk of complications and the need for nephrectomy. However, very few studies investigated the change in kidney volume after kidney transplantation.

**Material and methods:**

In this prospective cohort study, the change in native kidney volume in polycystic patients was evaluated with magnetic resonance imaging. Forty patients were included in the study. Kidney diameters and total kidney volume were evaluated with magnetic resonance imaging in patients who underwent simultaneous nephrectomy and kidney transplantation and in patients with kidney transplant alone, before transplantation and 1 year after transplantation.

**Results:**

There was a significant reduction of kidney volume after transplantation, with a mean degree of kidney diameters reduction varying from 12.24% to 14.43%. Mean total kidney volume of the 55 kidney considered in the analysis significantly reduced from 1617.94 ± 833.42 ml to 1381.42 ± 1005.73 ml (*P*<0.05), with a mean rate of 16.44% of volume decrease. More than 80% of patients had a volume reduction in both groups.

**Conclusions:**

Polycystic kidneys volume significantly reduces after kidney transplantation, and this would reduce the need for prophylactic bilateral nephrectomy in asymptomatic patients.

## Introduction

Autosomal dominant polycystic kidney disease (ADPKD) is a life-threatening genetic disorder characterized by the presence of fluid-filled cysts in various organs, primarily in the kidneys. ADPKD patients exhibit progressive cyst formation and massive renal enlargement that often leads to end-stage renal disease (ESRD) [1]. In recent years many efforts have been made to identify a predictive marker of disease progression and severity: total kidney volume (TKV) has been found to have a negative correlation with the glomerular filtration rate in patients with ADPKD [2,3], and TKV and the rate of kidney growth are strongly associated with the development of ESRD [2, 4–9].

Due to the progressive enlargement of kidney cysts, about 10–20% of patients with ADPKD will require a native nephrectomy due to cyst complications or to provide adequate space for kidney transplantation [10]. While native nephrectomy is clearly indicated in symptomatic patients (cyst bleeding, infection, cancer, early satiety), the indications for and timing of nephrectomy in ADPKD asymptomatic patients remains controversial, especially for those undergoing renal transplantation [11]. While is generally accepted that routine pre-transplant nephrectomy is no longer recommended [10, 12–19], and should be performed only when clearly indicated, the indications to mono- or bilateral nephrectomy is still debated. Bilateral nephrectomy with concurrent kidney transplantation has been suggested to reduce the incidence of post-transplant complications related to the retained kidney, such as bleeding, recurrent infections, and cancer. Although this procedure may be well tolerated with a high patient contentment, bilateral nephrectomy is associated with an increased risk of post-transplant complications [15–20]. Conversely, a unilateral nephrectomy with concurrent transplantation could be of benefit and might be sufficient to alleviate symptoms and to provide room for kidney transplantation [12–14, 21–27]. Moreover, the volume of native kidneys in patients with ADPKD significantly decreases after successful transplantation [28–30], reducing the need for a bilateral nephrectomy in asymptomatic patients.

Magnetic resonance imaging (MRI) examinations provide high-resolution information about the anatomic structure of the kidneys and are used to evaluate the TKV and the renal function in patients with ADPKD [2,3,7,31–35].

The aim of this study was to evaluate with MRI the change of kidney volume after kidney transplantation in patients with ADPKD. Moreover, this study evaluated also the change in kidney volume in patients undergoing simultaneous native nephrectomy and kidney transplantation, who are theoretically more prone to volume increase of remnant kidney.

## Materials and methods

In this single center, two-arm non-randomized study, from January 2000 to December 2016, 738 kidney transplants were performed (620 from deceased donors and 118 from living donors).

Of these, ADPKD was the cause of ESRD in 155 patients (21%). The diagnosis of ADPKD was made on the basis of medical history, physical examination, ultrasonography, computed tomography scan, magnetic resonance imaging (MRI) and in case of simultaneous nephrectomy, was confirmed by histologic examination [14]. The indication and surgical technique for native nephrectomy and for kidney transplantation have been described in details [14,27]. In brief, asymptomatic patients did not require pre-transplant native nephrectomy, and nephrectomy was performed at the time of transplantation only to create space for the kidney transplant, if indicated.

In order to investigate size variations of kidney, we enrolled patients for whom MRI reports were available. Patients were considered eligible to be included in the study if they have performed an abdominal MRI within six months before the transplantation, with the calculation of the diameters and the total kidney volume. All transplant patients underwent an evaluation of graft function with serum creatinine and graft sonography two days before the MRI.

**Fig 1:**
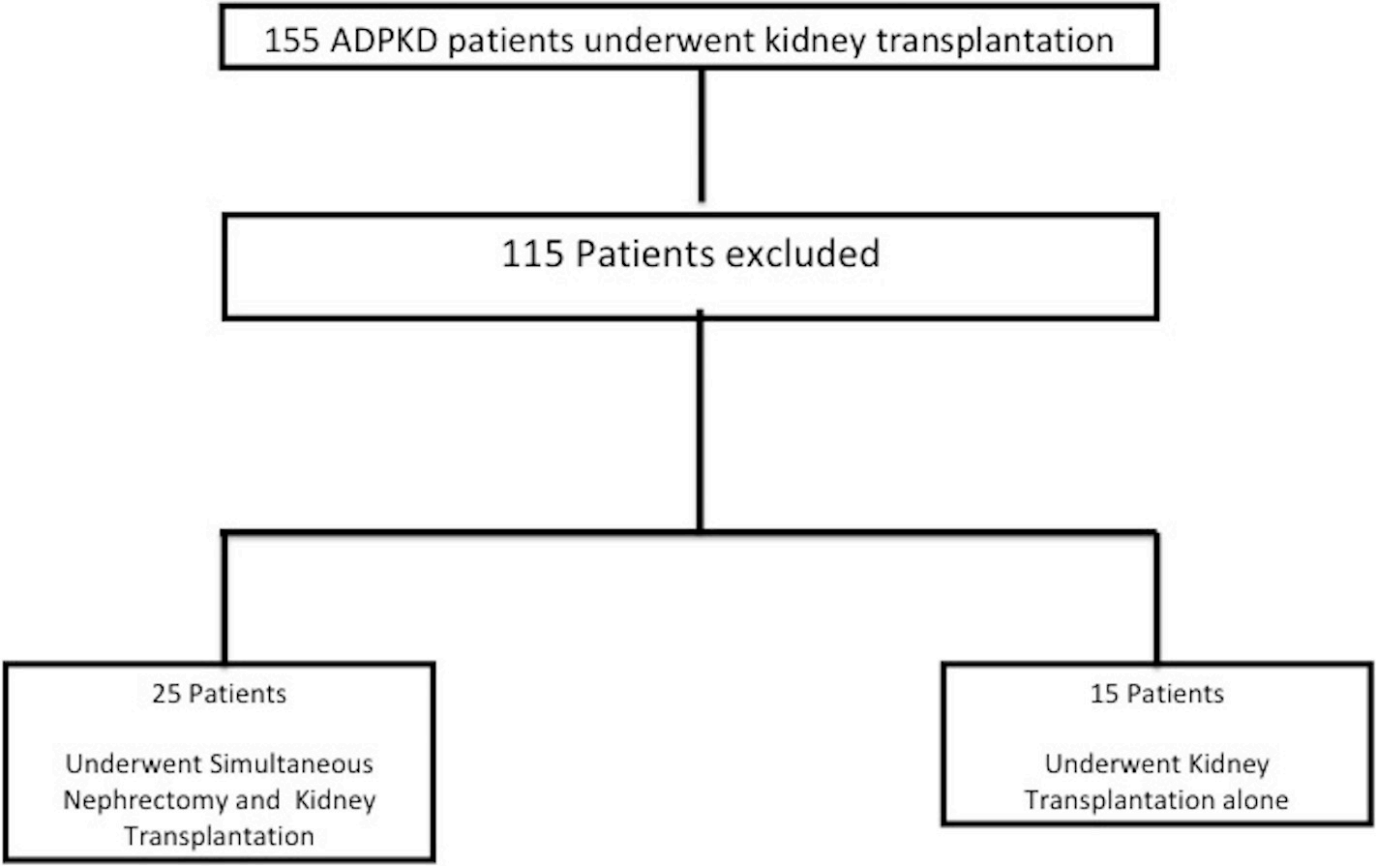
Flow chart of the study enrollment. Patients were excluded if they had only an image before, image was over six months before transplantation, underwent bilateral nephrectomy before transplantation, had concurrent cyst hemorrhage, infection or abscess, were unable to perform MRI, had less than 20 mL/min of eGFR at the time of last imaging, underwent combined transplantation and underwent second transplantation.

Forty patients fulfilled the inclusion criteria and were included in the study, and changes in the size of native kidneys after transplantation were analysed (Fig 1).

The change in kidney volume was evaluated in the whole group and then separately, for patients who had undergone simultaneous unilateral nephrectomy and kidney transplantation (SKT, 25 patients) and those who underwent the kidney transplantation alone (KT, 15 patients). Therefore, we analysed the change of bilateral (n = 30) and remaining unilateral (n = 25) kidneys volume separately. Magnetic Resonance Imagings were done one year after transplantation, in order to evaluate the total kidney volume and the presence of cysts complications (cancer, infection or asymptomatic bleeding). All MRI reports were reviewed by the same radiologist (SP).

All MRI examinations were performed using a 1.5 Tesla scanner. Two-dimensional SSFSE (Single Shot Fast Spin Echo) and FSPGR (Fast Spoiled Gradient Echo) were acquired in coronal and axial planes–including superior and inferior part of the abdomen; in addition, axial 2D SSFP (Steady State Free-Precession) T2/T1-weighted and axial three-dimensional FSPGR T1-weighted sequences were also performed. These axial planes were used for the evaluation of kidneys volume–so they were acquired with slice-thickness of 3–4 mm.

Due to the scarcity of data available in literature on post-transplant kidney volume measurement, we first evaluated the change in longitudinal diameters as previously reported [28] and the length, width, and depth (cranial-caudal, left–right, and anteroposterior dimensions, respectively) of each kidney were measured by multiplanar reformation of the T2-weigheted images data. Moreover, the total kidney volume was computed using the ellipsoid formula

*“Volume = p/6 x length x width x depth*, *with the width and depth measured at the renal hilum*.*”* [9,36].

The kidney and the cysts volumes of individual native kidneys were measured in millilitres, and the values from both kidneys were considered individually to yield the total kidney volume.

Kidney transplant recipients received a standard three-drug immunosuppressive therapy, with or without induction therapy with anti-interleukin-2 receptor antibodies (Simulect, Novartis, Basel, Switzerland) or with antithymocyte globulin (ATG-Fresenius, Fresenius, Bad Homburg, Germany), basing on both donor and recipient characteristics, as previously described [14]. The study was conducted in accordance with the principles of the 1975 Declaration of Helsinki and the Ethical Committee of the Azienda Policlinico of Catania ruled that no formal ethical approval was required in this particular case, as it conforms to normal clinical practice.

All patients signed an informed consent detailing all the procedures. The work has been reported in line with the STROBE criteria [37].

### Statistical analysis

Values are expressed as means ± standard error of the mean, unless otherwise stated. Comparisons of patient characteristics between groups were made using the continuity-adjusted chi-square test. Significance of differences between baseline and post-transplant values was tested by means of paired *t* test. Test were considered significant at *P* < 0.05. All statistical analyses were performed using SPSS (IBM SPSS Statistics for Windows, Version 22.0).

## Results

### Patient characteristics

The group of patients included 40 patients with ADPKD, who had undergone kidney transplantation. Thirty-five patients received the kidney from a deceased donor and five from a living donor. Twenty-five patients had undergone unilateral nephrectomy simultaneous to transplantation (SKT). One patient in the SKT group received dual kidney transplantation. Clinical characteristics of patients are reported in Table 1.

**Table 1:** Clinical data of 40 patients kidney transplant recipients. Patients of group SKT (25 patients) were compared with group KT(15 patients).

Characteristcs	Entire Study Group	SKT	KT	P Value SKT vs KT
**N**	40	25	15	
**Age (yrs)**	51.9 ± 9.43	52.3 ± 8.77	50.8± 11.35	.422
**Sex (M/F)**	24/16	17/8	8/7	.223
**BMI (Kg/m**^**2**^**)**	26.3 ± 4.3	27.4 ± 4.8	26.7 ± 5	.445
**Waiting List (months)**	18.2 ± 20.5	17.8 ± 18.8	19.4 ± 23.1	.835
**Pre-transplant dialysis (months)**	41.3 ± 39.5	33.8 ± 22.7	41.23 ± 44.2	.145
**Hemodialysis/peritoneal dialysis**		18/7	14/1	**< .05**
**Donor age (yrs)**	55.5 ± 14	52.8 ± 14.8	56.5 ± 12.1	.295
**Residual diuresis (ml)**	1124 ± 247	1235 ± 347	751.9 ± 143	**< .04**
**Indications to nephrectomy (n, %)**				
Creating space for graft positioning		23 (92)		
Recurrent urinary tract infections		0 (0)		
Recurrent hematuria		2 (8)		
**Cold ischemia time (min)**	922 ± 350.5	910 ± 340.8	932 ± 365.5	.643
**Operative Time (min)**	195 ± 75.2	198 ± 73	157 ± 48.4	**< .05**
**Native kidney longitudinal diameters at transplant (cm)**	20.08 ± 5.12	23.85 ± 4.33	16.77 ± 3.38	**< .01**
**Native kidney volumes at transplant (ml)**	1617 ± 833.42	1986.67 ± 752.24	833.13 ± 280.29	**< .01**
**Immunosuppression (n)**				
Tac/MMF/Ster	30 (75%)	19 (76%)	11 (73.3%)	.765
CyA/Ever/Ster	3 (7.5%)	2 (8%)	1 (6.6%)	.823
Tac/Ever/Ster	3 (7.5%)	2 (8%)	1 (6.6%)	.789
Ever/MMF/Ster	3 (7.5%)	2 (8%)	1 (6.6%)	.886
CyA/MMF/Ster	1 (2.5%)	0(0%)	1 (6.6%)	.992
**Mean serum creatinine**	1.92 ± 1.11	1.96 ± 1.13	1.87 ± 1.31	.442

The mean follow-up of patients was 6.3 years (range, 0.6–15.2 years). Recipient and donor characteristics were similar between the 2 groups (Table 1). Patients in the SKT group were more frequently receiving peritoneal dialysis, and had a lower time on dialysis and on the waiting list, without reaching the level of statistical significance. Moreover, there was no significant difference in renal function between the two groups at time of MRI examination. Five patients in the SKT group (20%) received their transplantation before dialysis initiation (pre-emptive).

In patients undergoing simultaneous nephrectomy, the mean weight of the removed kidneys was 2100 g (range, 820–5400 g). As expected, patients undergoing simultaneous nephrectomy had higher native kidney longitudinal diameters and volumes at transplantation compared with patients of group KT (*P*<0.01). Of interest, cold ischemia time, acute rejection episodes, hospital stay, and blood loss did not increase in patients undergoing simultaneous nephrectomy. Operative time was increased by 20–40 min depending on the size of the native kidney in the SKT group (P < .05), as previously reported [14]. No patient in the entire group died in the perioperative period.

There was 1 case of cystic bleeding, treated conservatively, while three patients suffered from cyst infection and required treatment. There was no cancer diagnosis in the remnant kidneys during the follow-up.

Two patients (5%), one in SKT and one in KT group, required a native nephrectomy to provide room for a second kidney transplantation 7 years after transplantation and for severe cysts infection, respectively.

### Changes in native kidney longitudinal diameters

Data of available MRI were used to evaluate the longitudinal diameters of 55 kidneys in 40 ADPKD patients. After transplantation, there was a highly significant reduction of kidney longitudinal diameters from 20.08 ± 5.12 cm to 17.48 ± 5.12 cm (*P*<0.01), with a mean rate of reduction of 13.34 ± 18.10%. There was a reduction of longitudinal parameters in 43 kidneys (78.2%), an increase in 11 kidneys (20%) and no size variations in one kidney (1.8%).

**Fig 2:**
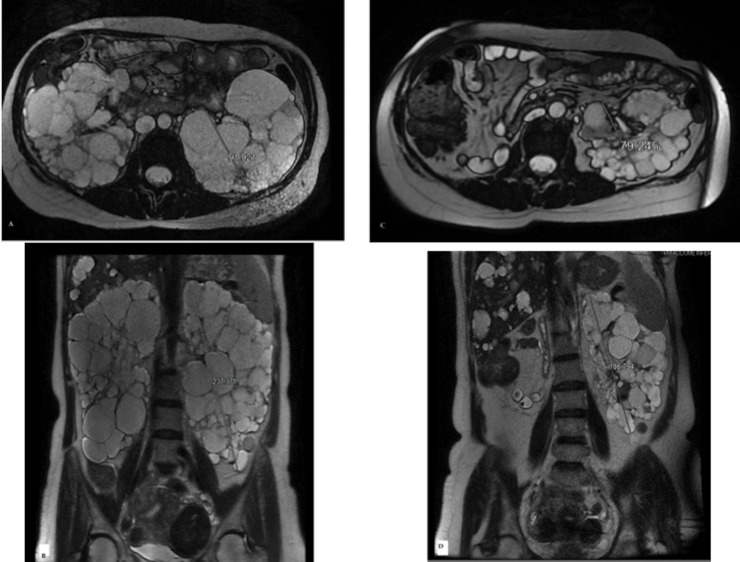
Magnetic resonance imaging of a 52 year-old woman, with end-stage renal disease due to autosomal dominant polycystic kidney disease. Pretransplant Axial T2 weighted (A) and Coronal T2 weighted images (B) demonstrated a massive enlargement of both kidneys with the lower pole in the iliac fossa. The patient underwent a kidney transplantation with simultaneous right nephrectomy, and 1-year Axial T2 weighted (C) and Coronal T2 weighted images (D) demonstrated a significant reduction of kidney volume.

Among the 25 recipients of SKT group, who underwent unilateral nephrectomy simultaneous to the transplantation, there was a significant reduction of longitudinal diameters of native kidneys from 23.85 ± 4.33 cm (range, 16–32 cm) to 20.09 ± 4.41 cm (range, 13.6–27.5 cm, *P*< 0.01), with a mean rate of longitudinal diameters decrease of 14.43± 18% (*P*< 0.01) (Fig 2).

**Fig 3:**
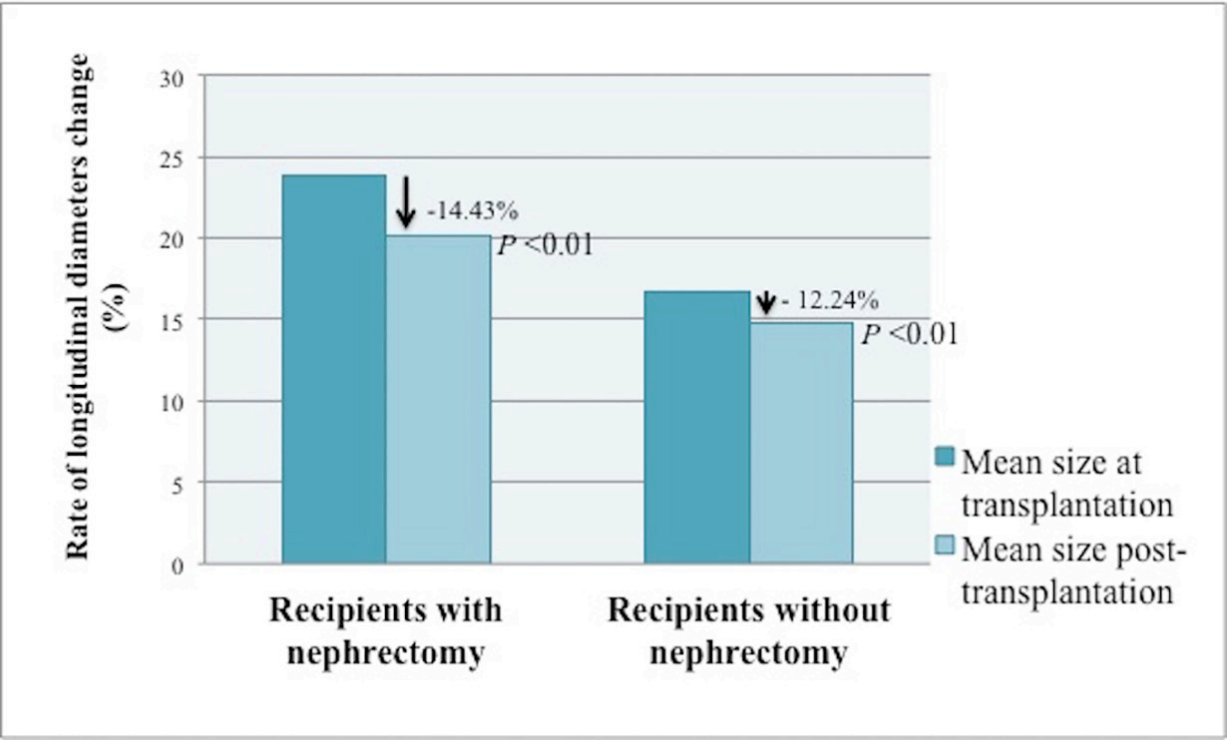
Rate of change in native kidney longitudinal diameters after transplantation. Patients with simultaneous nephrectomy and kidney transplantation were compared with patients who underwent a kidney transplant alone. Patients in both groups exhibited a statistically significant reduction of native polycystic kidney longitudinal diameters after transplantation, more pronounced in patients who underwent a simultaneous nephrectomy.

**Fig 4:**
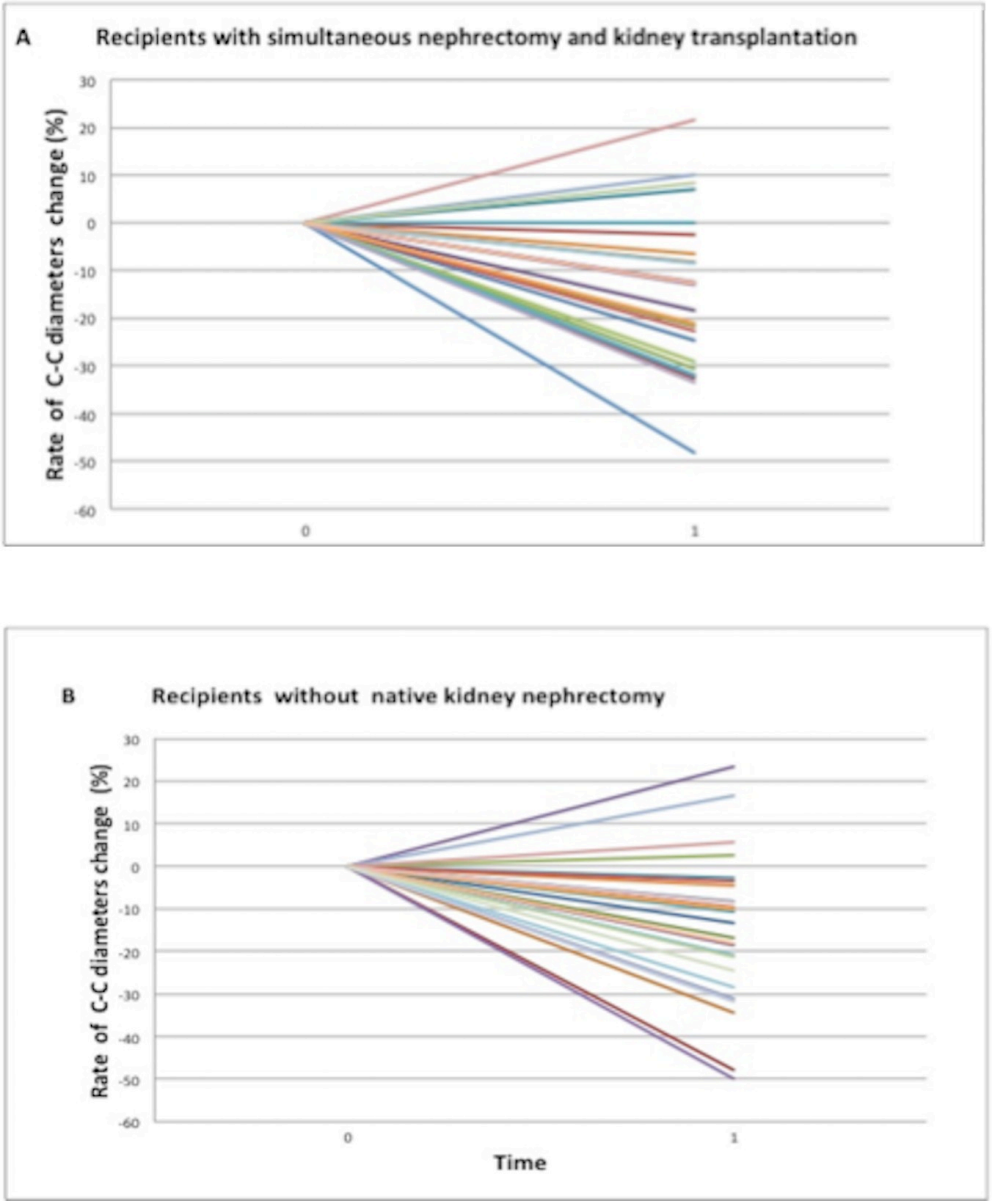
Visual figure demonstrating the rate of change in native kidney longitudinal diameters after transplantation compared to pre-transplant evaluation. The rates of change were equally statistically significant for patients who underwent simultaneous nephrectomy and kidney transplantation (A) and patients who underwent the kidney transplantation alone (B).

There was a reduction of diameters in 19 kidneys (76%), an increase in 5 kidneys (20%) and no size variation in one kidney (4%). In patients with longitudinal diameter reduction, the mean diameter reduced from 20.43 ± 5.12 cm at transplant to 16.04 ± 3.98 after transplantation. The reduction was highly significant (*P*<0.01). Therefore, this group exhibited a significant variation of kidney diameters after transplantation. **(**Figs 3 and 4A**).** In those patients with an increase of kidney diameters, the mean rate of increase was 11.8%.

These results were confirmed even in the 30 kidneys evaluated in the KT group: mean longitudinal diameter significantly reduced from 16.77 ± 3.38 cm (range, 9–19.5 cm) to 14.74 ± 4.27 cm (range, 6–24.3 cm, *P*< 0.01), with a 12.24±18.59% mean rate of longitudinal diameters decrease (*P*< 0.01). There was a reduction of diameters in 24 kidneys (80%) and an increase in 6 kidneys (20%). Again, polycystic kidneys significantly reduced after transplantation (Figs 3 and 4B). Moreover, although there was no significant difference in rate of kidney diameters reduction between the two groups, the SKT group displayed a more pronounced decrease of longitudinal diameters.

Trying to identify the clinical characteristics that could influence the volume reduction, patients with a decrease of kidney diameters (31 patients, 43 kidneys) were divided into three groups according to the degree of kidney reduction (Table 2).

**Table 2:** Patients with longitudinal kidney diameters decrease divided into 3 subgroups according to the rate of decrease. In patients with different variation in size of the two kidneys, it was considered the kidney with the highest variation.

Characteristics	< 15%	≥ 15 - < 30%	≥ 30%
**N (%)**	12 (38.8)	9 (29)	10 (32.2)
**Sex (M/F)**	5/7	5/4	5 / 5
**Age at transplant (yr),**	50.8 ± 6.54	54 ± 7.6	50.8 ± 10.81
**Pre-emptive transplant,(N, %)**	2 (16.6%)	1 (11.1%)	1 (10%)
**Pre.transplant dialysis (months)**	15.4 ± 10.87	18.33 ± 10.87	23.8 ± 10.59
**Nephrectomy/ Not nephrectomy, N (%)**	7 (58.3) / 5 (41.7)	6 (66.6) / 3 (33.4)	6 (60) / 4 (40)
**Mean Serum Creatinine (mg/dL)**	1.43 ± 0.68	1.64 ± 0.40	1.5 ± 0.43
**Mean e-GFR after transplant (mL/min/1.73 m**^**2**^**)**	64.71 ± 26.27	47 ± 18.77	56.87 ± 27.06

This analysis suggested that most patients (61.2%) exhibited a decrease of longitudinal parameters > 15% with a prevalence for SKT patients, and that patients with better renal function had the lowest degree of kidney reduction, although not statistically significant. Moreover, the degree of kidney diameters reduction was not influenced by recipient’s age, gender, type of pre-transplant dialysis, type of immunosuppression and time on dialysis.

### Changes in native kidney volume

Among the 55 kidneys (40 patients), after transplantation, kidney volumes reduced in 46 kidneys (83.6%), and increased in 9 kidneys (16.4%). There was a significant mean reduction of kidney volume from 1617.94 ± 833.42 ml to 1381.42 ± 1005.73 ml (*P*<0.05), with a mean rate of 16.44± 28.08% of volume reduction.

**Fig 5:**
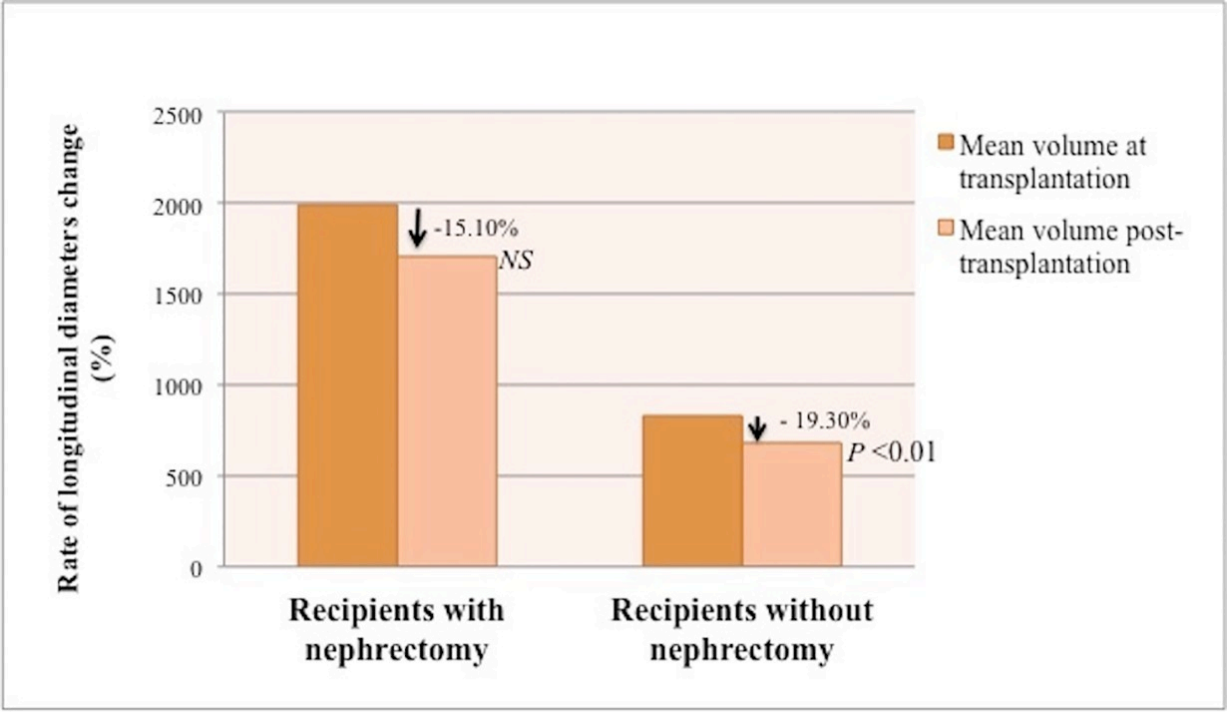
Rate of change in native kidney volume after transplantation in patients with ADPKD. Patients who did not undergo simultaneous nephrectomy displayed a highly significant reduction of kidney volume after transplantation, while patients who underwent simultaneous nephrectomy had a reduction of kidney volume, although not statistically significant.

**Fig 6:**
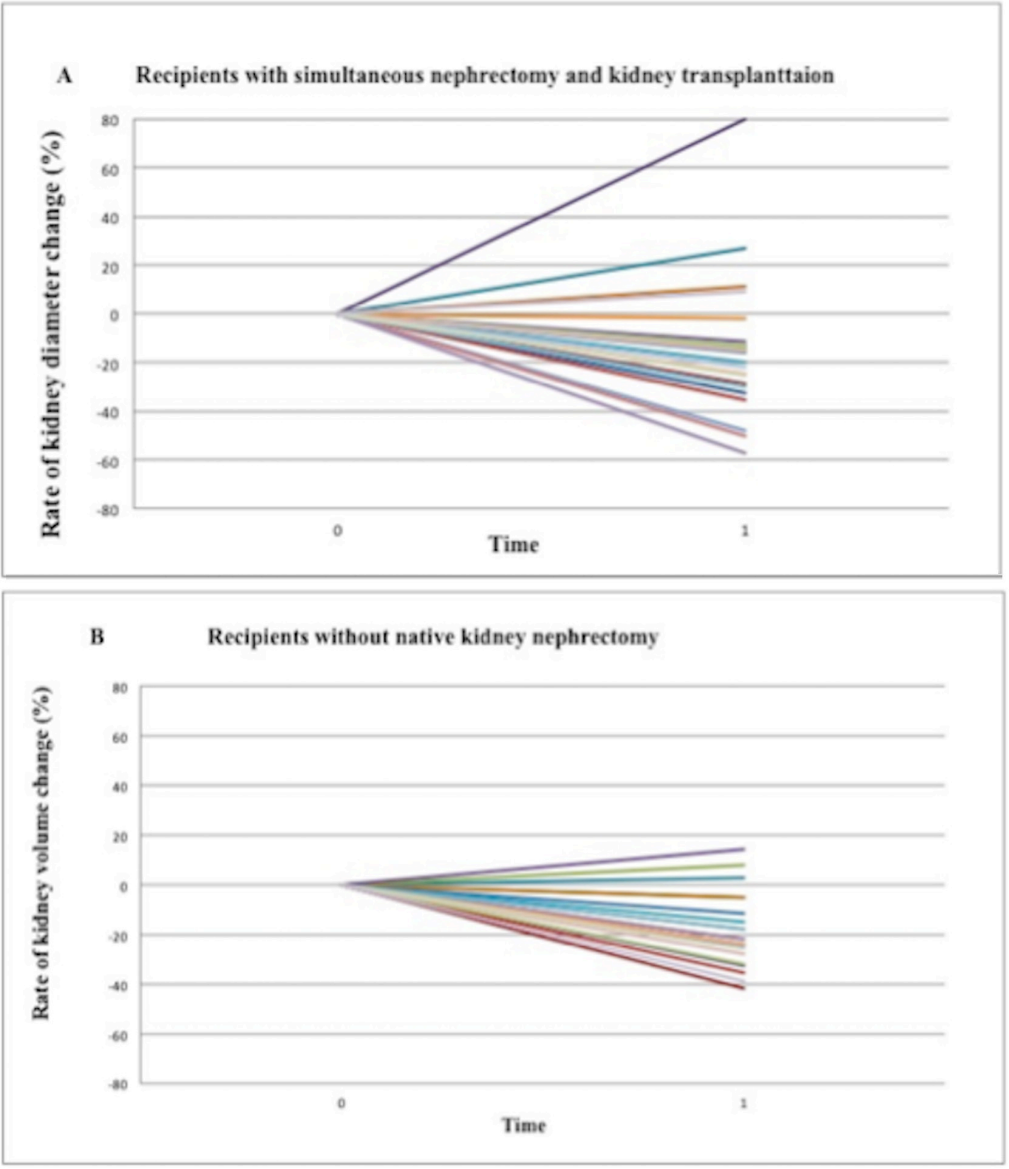
Visual figure of the rate of change of kidney volume after transplantation. Reduction of volume was less pronounced in patient with simultaneous nephrectomy (A) compared with patients without nephrectomy (B).

In the SKT group, kidney volume reduced after transplantation from 1986.67 ± 752.24 ml (range: 854.08 to 3315.84 ml) to 1709.89 ± 1064.02 ml (range: 508.46 to 2584.02 ml), with a mean rate of 15.10 ± 32.5% of volume reduction (*P* = 0.09). There were a reduction of volumes in 20 kidneys (80%) and an increase of kidney volumes in 5 kidneys (20%). **(**Figs 5 and 6A**).**

In the KT group, there was a significant reduction of kidney volume from 834.37 ± 236.98 ml (range: 544.26 to 1300 ml) to 683.42 ± 247.77 ml (range: 344.2 to 988.72 cm^3^) after transplantation (*P*< 0.01), with a mean rate of reduction of the 19.30±16.62%. There was a reduction of volumes in 26 kidneys (86.6%) with a mean rate of reduction of 25.28% and an increase in 4 kidneys (13.4%), with a mean increase of the 29.9%. Therefore, kidneys of this group exhibited even highly significant change in their volumes (Figs 5 and 6B**)**

When considering only kidneys with volume reduction, the results were more consistent: native kidneys volume reduced from 1562.98 ± 843.58 ml at transplant to 1144.89 ± 612.33 ml after transplantation (*P*<0.01).

The 33 patients displaying a volume reduction of polycystic kidneys were divided into three groups according to the degree of volume decrease (Table 3).

**Table 3:** Patients with total kidney volume decrease divided into 3 subgroups according to the degree of reduction. In patients with different variation in size of the two kidneys, it was considered the kidney with the highest variation.

Characteristics	< 15%	≥ 15 - <30%	≥ 30%
**N (%)**	13 (32.5)	6 (18.1)	14 (42.4)
**Sex (M/F)**	6/7	3/3	7/ 7
**Age at transplant (yr),**	51.3 ± 5.85	53.5 ± 6.7	52.3 ± 9.9
**Pre-emptive transplant,(N, %)**	2 (39.3%)	1 (16.6%)	1 (7.1%)
**Pre.transplant dialysis (months)**	14.7 ± 9.87	22.23 ± 9.87	22.9 ± 11.60
**Nephrectomy/ Not nephrectomy, N (%)**	8 (61.5) / 5 (39.5)	4 (66.6) / 2 (33.4)	8 (57.1) / 6 (43.9)
**Mean Serum Creatinine (mg/dL)**	1.38 ± 0.65	1.52 ± 0.38	1.4 ± 0.39
**Mean e-GFR after transplant (mL/min/1.73 m**^**2**^**)**	63.71 ± 22.27	45 ± 19.76	55.3 ± 22.06

Again, patients with the lowest degree of volume reduction more frequently received a pre-emptive transplantation, had a better renal function and underwent a simultaneous nephrectomy, without reaching the statistical significance. Moreover, most patients (60.5%) had a volume reduction > 15% and the degree of volume reduction was not significantly influenced by recipient’s age, gender, type of pre-transplant dialysis, and time on dialysis.

## Discussion

This study demonstrated that the kidney volume in patients with ADPKD significantly decreases after kidney transplantation.

One of the major issues in the management of patients with ADPKD is to find a reliable marker of progression of disease toward the end-stage renal disease and the need for kidney transplantation. In recent years, the total kidney volume has emerged as a reliable and accurate method to evaluate the degree of renal function and higher rates of kidney enlargements are associated with a more rapid progression to renal failure [2–9].

The TKV may be measured in different ways, including imaging techniques utilizing MRI and computed tomography (CT). CT scan may be more readily accessible and has been previously used to evaluate the change in polycystic kidney volume after kidney transplantation [28], but its use may be limited for the radiation exposure and the need for iodinated contrast [8,38]. Otherwise, MRI demonstrated a greater precision and MRI measurements of renal cysts and volume are reliable and accurate [32,33,38], with minimal bias and lower inter- and intraoperator variability [8,38].

Total kidney volume may be measured using stereology techniques, in which area measurements and slice thickness of a series of contiguous images are used to determine the kidney volume [38–40]. Although this method yielded reliable results for kidney and cyst volumes [38], it requires 45 to 55 minutes to complete, and its accuracy may be limited by display window setting and grid size [9,31], so that this approach is no longer recommended in clinical practice [40]. In this study, we evaluated using MRI, the change in kidney volume in patients with ADPKD before and after the kidney transplantation. The kidney volume was calculated using the ellipsoidal formula, that requires only 5 to 7 minutes to complete [9,31] and has a good correlation with CT/MRI stereology measurements, without systematic overestimation or underestimation [41].

The progressive kidney enlargement may increase the risk of cysts complications, including gross hematuria, infection and cancer, demanding the need for nephrectomy [38], in patients scheduled for kidney transplantation. However, the choice for unilateral vs. bilateral nephrectomy is still debated. Bilateral nephrectomy with concurrent kidney transplantation is associated with an increased risk of post-transplant complications [15–19, 42,43]. Leaving a kidney in situ may, on the other hand, potentially increase the risk of cyst complications, due to the progressive enlargement of kidney volume. However, the rate of complications in the retained kidney, including cancer, seems low [14], so that unilateral nephrectomy with concurrent transplantation might be sufficient to alleviate symptoms and to provide room for kidney transplantation in asymptomatic patients [14].

In principle, knowing the evolution and the changes in size of polycystic kidney volume after kidney transplantation might help in the choice of the surgical approach [44]. However, data on literature demonstrating the effects of renal transplantation on native kidney volume are very poor, although the reduction of blood flow mediated by the use of calcineurin inhibitors has been suggested as a possible mechanism [28].

In their study, Yamamoto et al. [28] used CT imaging to determine total native kidney volume before and after kidney transplant in 33 patients. This study demonstrated that bilateral kidney volumes tended to gradually increase during the 6-month period immediately before transplantation, and these data are consistent with the direct correlation between the increase of TKV and the worsening of kidney function [2–9]. Indeed, CT scans performed 1 and 3 years after transplantation, showed a significant decrease of native kidneys volume. Interestingly, patients with the highest rate of volume decrease (>38%) were younger, but no other factors, such as gender, dialysis vintage, serum creatinine, estimated GFR, calcineurin inhibitor (cyclosporine vs. tacrolimus), and native kidney volume at transplant were significantly associated with the degree of kidney decrease [28].

However, recent studies found that the rate volume reduction may be influenced by a shorter time on dialysis and higher GFR [29], although the type of immunosuppression does not influence the rate of volume reduction [28–30], as reported in our study.

In this study we prospectively evaluated the volume of the remaining polycystic kidneys before and one year after kidney transplantation using a MRI technique. We evaluated 40 patients with ADPKD and kidney transplantation, and change in kidney diameters and volumes were analysed separately between patients who had undergone simultaneous nephrectomy and those who did not. This study demonstrated that there is a significant reduction of kidney diameters after kidney transplantation, more pronounced in patients of the SKT group. Although this finding is not completely confirmed when considering the variation of the total kidney volume, this observation is surprising giving that unilateral nephrectomy may theoretically lead to the hypertrophy of the existing kidney and an increase in glomerular filtration rate as it compensates for the loss of the other kidney [45], and the unilateral native kidney may tend to occupy the space left by the removed kidney. However, the remaining kidney progressively looses its function after the restoration of normal functionality with transplantation and this may explain the significant reduction of kidney volume after successful kidney transplantation observed in this study.

The mean degree of kidney diameters reduction varied from 14.43% in SKT group to 12.24% in KT group, but the 61.2% of kidneys displayed a reduction >15%.

When considering the kidney volume, the results were more impressive: mean total kidney volume of the 55 kidney considered in the analysis significantly reduced from 1617.94 ± 833.42 ml to 1381.42 ± 1005.73 ml (*P*<0.05), with a mean rate of 16.44% of volume decrease. More than 80% of patients had a volume reduction in both groups, with a mean rate of reduction of the 19.30% in the KT group. Again, the 60.5% of patients had a volume decrease > 15%.

The results of this study may have some important practical applications: the reduction of the kidney volume after transplantation may make unnecessary the bilateral nephrectomy if the space for kidney graft is available in the absence of infection, bleeding, or malignancy [14,28,29]; moreover, the reduction of kidney volume after transplantation may reduce, in principle, the risk of complications related to the enlargement of kidney cysts, such as gross hematuria and hypertension [8]. This was confirmed by our previous study [14], which demonstrated that only a minority of ADPKD patients would require a native nephrectomy after transplantation, most often to allow the space for a second transplantation. In this study only the 5% of patents required a native nephrectomy after the transplantation. Moreover, the risk of finding an incidental renal neoplasm is not different in ADPKD patients compared with the general population [46].

Although the study reported such important findings, we are conscious of its limitations. Although similar findings has been already reported in previous studies [28], this study used a more accurate imaging method (MRI) to evaluate the change in kidney volume and the sample size is larger; the measurement of diameters and kidney volume may have a great variability: however, all pre-transplant and post-transplant procedures were performed by the same radiologists with the same MRI protocol, reducing the bias of intra-operator variability. The variation of kidney volume was evaluated only one year after transplantation and we are not able to evaluate the long-term variation of kidney volumes: however, previous studies [28,29] demonstrated that the highest decrease of kidney volume is observed during the first year after transplantation, and that the volume slowly changed during the subsequent years; considering the high costs of MRI, performing further annual studies in asymptomatic patients was considered unreliable. The study sample is relatively small: however, this is the largest series in literature comparing the variation of kidney volume after kidney transplantation in patients undergoing simultaneous nephrectomy or kidney transplant alone, and the difficulty to have a MRI imaging immediately before transplantation, significantly limits the number of patients potentially eligible for this study; however, it could provide a significant evidence that the reduction of native kidneys was observed after transplantation.

## Conclusions

This MRI study clearly demonstrated that polycystic kidneys volume significantly decrease after kidney transplantation, even after simultaneous nephrectomy. This would reduce the need for bilateral nephrectomy in asymptomatic patients, in whom a unilateral nephrectomy for providing space at the time of kidney transplantation, if indicated, could be considered the preferred approach.

## Abbreviations

ADPKD: Autosomal Dominant Polycystic Kidney Disease
ESRD: End-stage Renal Disease
TKV: Total Kidney Volume
MRI: Magnetic Resonance Imaging
CT: Computed Tomography
Declarations of interest: none
